# Association of Socioeconomic Status with Overall and Cause Specific Mortality in the Republic of Seychelles: Results from a Cohort Study in the African Region

**DOI:** 10.1371/journal.pone.0102858

**Published:** 2014-07-24

**Authors:** Silvia Stringhini, Valentin Rousson, Bharathi Viswanathan, Jude Gedeon, Fred Paccaud, Pascal Bovet

**Affiliations:** 1 Institute of Social and Preventive Medicine, Lausanne University Hospital, Lausanne, Switzerland; 2 Ministry of Health, Victoria, Republic of Seychelles; Universidade Federal do Acre (Federal University of Acre), Brazil

## Abstract

**Background:**

Low socioeconomic status (SES) is consistently associated with higher mortality in high income countries. Only few studies have assessed this association in low and middle income countries, mainly because of sparse reliable mortality data. This study explores SES differences in overall and cause-specific mortality in the Seychelles, a rapidly developing small island state in the African region.

**Methods:**

All deaths have been medically certified over more than two decades. SES and other lifestyle-related risk factors were assessed in a total of 3246 participants from three independent population-based surveys conducted in 1989, 1994 and 2004. Vital status was ascertained using linkage with vital statistics. Occupational position was the indicator of SES used in this study and was assessed with the same questions in the three surveys.

**Results:**

During a mean follow-up of 15.0 years (range 0–23 years), 523 participants died (overall mortality rate 10.8 per 1000 person-years). The main causes of death were cardiovascular disease (CVD) (219 deaths) and cancer (142 deaths). Participants in the low SES group had a higher mortality risk for overall (HR = 1.80; 95% CI: 1.24–2.62), CVD (HR = 1.95; 1.04–3.65) and non-cancer/non-CVD (HR = 2.14; 1.10–4.16) mortality compared to participants in the high SES group. Cancer mortality also tended to be patterned by SES (HR = 1.44; 0.76–2.75). Major lifestyle-related risk factors (smoking, heavy drinking, obesity, diabetes, hypertension, hypercholesterolemia) explained a small proportion of the associations between low SES and all-cause, CVD, and non-cancer/non-CVD mortality.

**Conclusions:**

In this population-based study assessing social inequalities in mortality in a country of the African region, low SES (as measured by occupational position) was strongly associated with overall, CVD and non-cancer/non-CVD mortality. Our findings support the view that the burden of non-communicable diseases may disproportionally affect people with low SES in low and middle income countries.

## Introduction

In high income countries, low socioeconomic status (SES) consistently predicts higher adult mortality for most causes of death [1]–[3]. This issue remains largely unexplored in low and middle income countries (LMIC) because of limited availability of reliable mortality data. In addition, although the few studies examining social differences in mortality in LMICs have generally reported an inverse association between SES and mortality, some studies have found higher mortality in the higher SES groups.

An inverse association between education and all-cause mortality has been observed in rural Bangladesh [4], and between occupational status and all-cause mortality in Sao Paolo, Brazil [5]. In rural south India, low SES individuals had a higher incidence of mortality due to all causes in all age groups [6], while the Indian Human Development Study showed that a low income was associated with a higher mortality burden [7]. In China, each additional year of school was associated with a 5% reduction in mortality among elderly men and women [8]. In the Beijing Multi-dimensional Longitudinal Study on Aging, people with high vs. low SES (according to several indicators) had a greater life expectancy [9]. One study found higher mortality among the least educated in several countries in Latin America, India and China [10]. In the African region, one study using a demographic surveillance system in Ethiopia reported lower survival among those with lower compared to higher literacy levels [11], and in two different South African studies low SES was related to a higher adult mortality risk [12], [13]. On the contrary, in a rural South African community SES was not associated with adult mortality [14], and a longitudinal study of elderly Costa Ricans showed increasing mortality with higher levels of education and wealth [15].

If studies in LMICs generally show a greater overall mortality among the most disadvantaged SES groups, fewer studies have assessed the social patterning of cause-specific mortality. Given the higher burden of communicable diseases among the most disadvantaged (with the possible exception of HIV/AIDS [16]), it is reasonable to assume that low SES individuals also share a higher mortality burden for these diseases. However, the issue is more complex for non-communicable diseases (NCDs), as the social distribution of NCDs and their risk factors is part of the health transition, is related to socioeconomic development, and as such may change over time [17]–[19].

A limited number of studies have examined socioeconomic differences in cause-specific mortality among adults in LMICs. In rural south India, people with a low SES had higher mortality for all specific causes of death (including for infectious diseases, cancer, cardiovascular diseases and respiratory diseases) [6]. However, in another Indian study [20], although a strong educational gradient in cardiovascular mortality was observed among people who could read and write, people who could not read and write (illiterates) had lower cardiovascular mortality than those with primary or middle education. Finally, a recent meta-analysis showed that mortality was overall higher in low vs. high SES individuals in low and middle income Asian countries [21]. With regards to Latin America, a report from the 1990s in Sao Paolo, Brazil, showed higher cancer mortality among people with low education, with the exception of lung cancer for which an opposite pattern was observed [22]. A recent Colombian study showed higher mortality in people with low vs. high education for all-specific causes of death examined, including NCDs and injuries [23].

Several factors have been proposed to explain social inequalities in mortality, including SES differences in several domains such as lifestyle factors, social norms, physical living and working environments, health education, health consciousness, attitude and motivation, and access to and utilization of health care [24]–[26]. Recent evidence suggests a prominent role of SES differences in lifestyle factors in explaining social inequalities in chronic disease incidence and mortality [27]–[29]. To our knowledge, no study has so far examined the extent to which lifestyle-related risk factors explain social inequalities in mortality in a LMIC.

The main objective of this study is to examine SES differences in both overall and cause-specific mortality in the Republic of Seychelles, a small island state in the African region. Additionally, this study explores the extent to which social inequalities in mortality are explained by socioeconomic variations in the prevalence of common risk factors for chronic diseases.

## Data and Methods

### Study population

The Republic of Seychelles is a rapidly developing small island state in the Indian Ocean (African region), located east of Kenya and north of Mauritius. The population size was 67,000 in 1989 (44% aged≥25 years) and 84,000 in 2004 (57% aged≥25 years). The majority (>80%) of the population is of African descent. Life expectancy at birth increased from 63 to 69 years in men and from 73 to 76 years in women between 1989 and 2004 [30]. The gross domestic product (GDP) per capita rose, in real terms, from $2927 in 1980 to US$ 5239 in 2004. Health care, including access to medications, has been available with no fee to all inhabitants through a national health system during the whole study period. The prevalence of several lifestyle-related risk factors was high in Seychelles as early as in the late 1980s, with risk factors decreasing over time (particularly smoking), plateauing (blood pressure, blood cholesterol) or increasing (mainly obesity and diabetes) [31]–[34]. CVD has been the leading cause of mortality in Seychelles since the late 1980s, but age-adjusted rates have decreased substantially between 1989 and 2010 [35].

Three independent population-based examination surveys of lifestyle-related risk factors were conducted in 1989, 1994 and 2004. Participation was voluntary and participants gave informed consent. In 1989 and 1994, verbal consent was obtained in view of the large number of illiterate participants. In 2004, written consent was obtained. All surveys were approved by the Ministry of Health's Health Research and Ethics Committee. The committee approved the consent procedure. The sampling frames, methods and main results of the three surveys have been described previously [36]–[38]. Briefly, each survey consisted of an age- and sex-stratified random sample of the total population aged 25-64 years. Inclusion criteria were unchanged in the three surveys. For each survey, eligible participants were selected from an electronic database derived from population censuses, regularly updated on a yearly basis by civil status authorities. The surveys were attended by 1081 persons in 1989 (86.4% participation rate), 1067 in 1994 (87%), and 1255 in 2004 (80.2%). A total of 1585 men and 1818 women participated in the three surveys. In all surveys, trained officers administered a structured questionnaire on demographic and lifestyle factors to the participants, using similar questions. Analyses were based on 3246 participants with complete data on all risk factors considered for the study. Data are available from the authors.

### Measures

#### Mortality

The vital status of all survey participants was ascertained by linkage with deaths registries for the period 1989–2012. All deaths occurring in Seychelles are medically certified using death certificates as recommended by the World Health Organisation (http://www.who.int/classifications/icd/ICD-10_2nd_ed_volume2.pdf). Information for each field is registered into a central database as entered by the certifying doctors. For the sake of internal consistency in causes of deaths over the 23-year period of the study, this raw textual information this raw textual information was therefore reviewed and recoded and the underlying cause of death was selected using WHO rules [35], [39]. The *International Classification of Diseases, 10^th^ Revision* (ICD-10) was used to define cancer (C00-C97) and cardiovascular disease (CVD, I00-I99) mortality. In this study, the category “Non-cancer/non-CVD mortality” includes all remaining deaths not classified as cancer or CVD. This category includes various causes of death, particularly infectious diseases and external causes of death.

#### Measurement of cardiovascular risk factors (CVRF)

*Smoking* was defined as smoking at least one cigarette every day. Alcohol intake was assessed from a set of questions on drinking frequency and volume for the six main alcoholic beverages (beer, wine/liquor, spirits and locally made homebrews), taking advantage of the fact that only a limited number of brands and contents were available in the country up to 2004 [40]. Mean daily ethanol intake per week was calculated. *Heavy drinking* was defined as consuming more than 75 g of ethanol per week.

Weight was measured with calibrated medical electronic scales (Seca) and height was measured using fixed stadiometers. Body mass index (BMI) was calculated as weight divided by height squared (kg/m^2^). *Obesity* was defined as BMI≥30. Blood pressure (BP) was measured with a mercury sphygmomanometer using a cuff adapted to the arm circumference and was based on the two last of three readings taken at intervals of at least two minutes, after the participants had been quiet in the study center for at least 30 minutes and seated for >10 minutes. *Hypertension* was defined as BP≥140/90 mmHg or taking treatment.

Fasting blood was collected in all three surveys the early morning after an overnight fast, blood was spun at the study centers, and serum was immediately frozen to −20°C. All analyses, except for capillary glucose, were performed at university laboratories in Switzerland. In 1989 and 1994, total cholesterol was measured enzymatically (CHOD-PAP method) using reagents from Boehringer (Manheim, Germany). In 2004, blood lipids were measured using a Hitachi 917 instrument and Roche reagents. *High total cholesterol* was defined as total cholesterol ≥6.2 mmol/l (240 mg/dl). Fasting blood glucose (FBG) was determined immediately after blood drawing using point-of-care instruments in 1989 and 2004. In 1989, venous blood glucose was measured using a reflectance meter (Reflomat with Hemoglucotest reagent strips, Boerhinger), a validated and frequently used glucometer at the time. In 1994, presence of sugar in the urine was tested in all participants using dipsticks (Glukotest, Boehringer, Mannheim, Germany). In 2004, glucose was measured on venous blood using a Cholestec LDX analyzer (Cholestec, Hayward, USA), a reliable alternative to conventional laboratory devices. *Diabetes* was defined as FBG≥7.0 mmol/l (126 mg/dl) (1989, 2004) or positive glucosuria or history of diabetes (1994) [41].

#### Socioeconomic status (SES)

In all three surveys, the same question classified occupation in six categories, based on the participant's current occupation or his/her past occupation if a participant was not currently employed. More than 80% of participants were currently employed at each survey. Since participants were aged less than 65 years at baseline, and in view of high employment rate in Seychelles for both sexes, a large majority of persons could report a current or recent occupation. In rare occurrences of participants who declared to have never worked, they were categorized as “non-qualified”. In this paper, we grouped the 6 categories into three categories. The highest category includes “professionals” and “skilled non manuals”, the intermediate category includes “semi-skilled non-manuals”, “skilled manuals”, and “semi-skilled manuals” and the lowest category includes “unskilled workers” and “non-qualified” [34].

### Statistical analysis

In a preliminary analysis, we tested whether there was a modification effect by gender in the association between SES and mortality, and found no evidence for such an effect (*p* for interaction  = 0.560). Men and women were thus analyzed together and all analyses adjusted for sex. Age- and sex- standardized mortality rates per 1000 person-years were calculated for all-cause, CVD, cancer, and non-cancer/non-CVD mortality. The associations between SES and mortality and between other risk factors and mortality were assessed using Cox proportional regression analysis with age as the time scale. Participants who were still alive at the end of follow-up were censored at December 31^st^, 2012. Participants were considered at risk of dying in our analyses only from the age they reached when they were enrolled into the study (i.e. from their age in 1989, 1994 or 2004). Participants for whom no certificate of death could be found were considered as alive and were censored on December 31^st^, 2012. To estimate the baseline survival function (referring to the participants with all covariates equal to zero in a regression model), we used the Breslow's method [42]. Since the youngest participants included into our study were 25 years old, estimates of survival are conditional on having reached the age of 25 years.

The Cox regression model for the association between SES and mortality outcomes was first adjusted for sex and year of birth (model 1). Subsequently, the model was further adjusted for modifiable risk factors one at a time and then simultaneously. The contribution of each risk factor to the SES-mortality association was determined by the percentage reduction in the coefficient for SES after inclusion of the considered risk factor, using the formula: 100 * (Model 1 −Model 1_risk factor_)/(Model 1) [29], [43]. Although this approach (“difference method”) may provide biased estimates under some circumstances, particularly when the outcome is frequent [44], [45], in our study this potential problem was limited by the relative low frequency of our health outcome (mortality), by the absence of exposure-mediator interaction (all p values for interaction between SES and risk factors >0.05), and by controlling for potential mediator-outcome confounders (age and gender).

The proportional hazard assumption for Cox regression models assessed using Schoenfeld residuals was not violated. Analyses were performed using Stata 12.1 (Stata- Corp, College Station, Texas). The graphical display of the results was produced using R (R Project for Statistical Computing version 2.5.1).

## Results

During the follow-up period (0–23 years; mean 15.0 years), 523 participants died (mortality rate 10.8 per 1000 person-years). The main causes of death were CVD (219 deaths) and cancer (142 deaths) (**Table 1**). Participants in the low vs. high SES groups were more likely to be smokers, heavy drinkers, and obese (*p*<0.001). The prevalence of diabetes and hypertension was similar across socioeconomic categories (*p*>0.05), while high cholesterol was more prevalent in the high vs. low SES group (*p* = 0.048).

**Table 1:** Characteristics of the participants included in the study by socioeconomic status.

	Socioeconomic status				
	High	Middle	Low	*P* a	Overall
N (%)	474 (14.6)	1481 (45.6)	1292 (39.8)		**3246**
Mortality, N (Rate)	32 (7.1)	221 (10.1)	270 (12.9)	0.046	**523 (10.8)**
Cardiovascular, N (Rate)	11 (2.4)	91 (4.3)	117 (5.3)	0.324	**219 (4.5)**
Cancer, N (Rate)	11 (2.8)	57 (2.5)	74 (3.6)	0.218	**142 (2.9)**
Non-cancer/Non-CVD, N (Rate)	10 (1.9)	77 (3.4)	84 (4.2)	0.013	**171 (3.5)**
Smoking, N (%^b^ **)**	55 (17.7)	360 (21.9)	329 (26.0)	<0.001	**744 (22.9)**
Heavy drinking, N (%^b^ **)**	21 (7.3)	182 (11.1)	190 (15.0)	<0.001	**393 (12.1)**
Obesity, N (%^b^ **)**	79 (16.3)	276 (19.9)	319 (23.4)	0.001	**674 (20.8)**
Diabetes, N (%^b^ **)**	22 (7.9)	133 (8.9)	144 (10.0)	0.159	**299 (9.2)**
Hypertension, N (%^b^ **)**	178 (46.5)	663 (45.8)	642 (45.2)	0.560	**1483 (45.7)**
High cholesterol, N (%^b^ **)**	125 (28.4)	367 (26.4)	351 (24.5)	0.048	**843 (26.0)**

SD: Standard Deviation; Rate: Age- and sex- adjusted mortality rate per 1000 person-years (mean follow-up for mortality 15.0 years).

a *p* for linear trend across socioeconomic categories.

b Age- and sex-adjusted prevalence. Heavy drinking is defined as consuming ≥75 g of ethanol per week; obesity as body mass index≥30 kg/m^2^; diabetes as fasting blood glucose ≥7.0 mmol/l (126 mg/dl) (1989, 2004) or positive glucosuria or history of diabetes (1994); hypertension as blood pressure≥140/90 mm Hg; high cholesterol as total cholesterol ≥6.2 mmol/l (240 mg/dl).

Tobacco use and heavy drinking were strongly associated with mortality from all-causes, cancer and non-cancer/non-CVD (**Table 2**). Obese participants had a higher risk of CVD mortality and participants with diabetes were at higher risk of mortality from all-cause, CVD and for non-cancer/non-CVD mortality. Hypertension was associated with all-cause and CVD mortality.

**Table 2:** Association of selected risk factors with all-cause and cause specific mortality among men and women (N = 3246).

	MORTALITY							
	All-causes		Cancer		CVD		Non cancer/non CVD	
Risk factors	HR^a^	95% CI	HR^a^	95% CI	HR^a^	95% CI	HR^a^	95% CI
Smoking	1.66	1.37–2.02	2.47	1.70–3.60	1.33	0.98–1.81	1.58	1.13–2.20
Heavy drinking^b^	1.52	1.25–1.92	1.84	1.24–2.73	1.20	0.84–1.72	1.73	1.22 –2.47
Obesity^b^	1.23	0.98–1.55	0.76	0.46–1.27	1.80	1.30–2.48	0.90	0.56–1.42
Diabetes^b^	1.75	1.40–2.19	1.02	0.61–1.70	2.00	1.45–2.77	2.06	1.40–3.01
Hypertension^b^	1.35	1.12–1.62	1.05	0.74–1.49	1.93	1.41–2.63	1.09	0.79–1.49
High cholesterol^b^	0.92	0.76–1.13	0.76	0.52–1.15	1.07	0.80–1.43	0.67	0.49–0.91

CI: Confidence Interval; CVD: Cardiovascular Disease; HR: Hazard Ratio.

a Sex and year of birth-adjusted.

b Heavy drinking is defined as consuming ≥75 g of ethanol per week; obesity as body mass index ≥30 kg/m^2^; diabetes as fasting blood glucose ≥7.0 mmol/l (126 mg/dl) (1989, 2004) or positive glucosuria or history of diabetes (1994); hypertension as blood pressure≥140/90 mm Hg; high cholesterol as total cholesterol ≥6.2 mmol/l (240 mg/dl).

**Figure 1:**
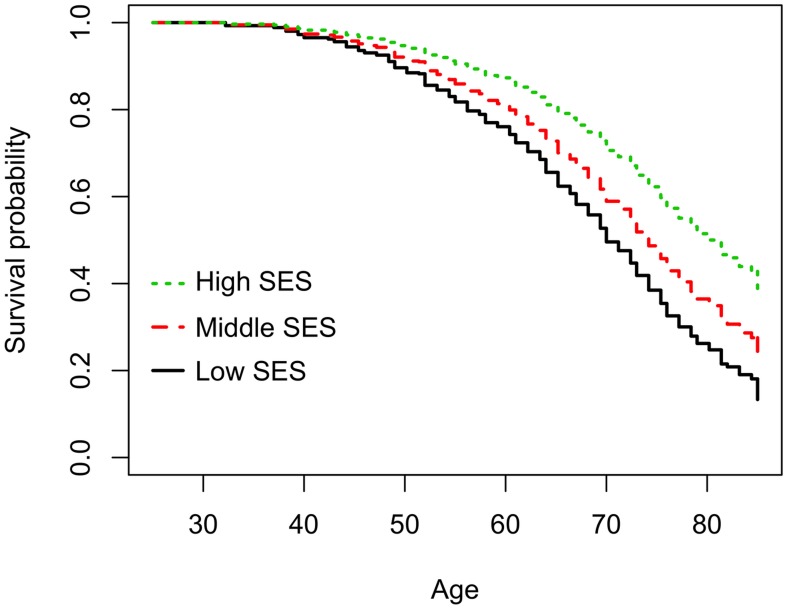
Survival probability from the age of 25 years by socioeconomic category.

Participants in the low SES group had an 80% increased risk of dying compared with participants in the high SES category (95%CI:1.24; 2.62) (**Table 3** and **Figure 1**). Smoking was the single largest contributing factor, and all risk factors combined explained about one fourth of the association between SES and all-cause mortality.

**Table 3:** Socioeconomic differences in all-cause mortality and contribution of modifiable risk factors (N = 3246, deaths = 522).

	SOCIOECONOMIC STATUS				
	High	Middle		Low	
	HR (95% CI)	HR (95% CI)	% Δ	HR (95% CI)	% Δ
**ALL-CAUSE MORTALITY**					
Model 1^a^	1.00	1.45 (1.00–2.10)		1.80 (1.24–2.62)	
Model 1 + smoking	1.00	1.37 (0.94–1.99)	*−15*	1.64 (1.13–2.39)	*−16*
Model 1 + heavy drinking^b^	1.00	1.41 (0.97–2.05)	*−7*	1.68 (1.16–2.44)	*−12*
Model 1 + obesity^b^	1.00	1.43 (0.94–2.08)	*−4*	1.81 (1.24–2.62)	*0*
Model 1 + diabetes^b^	1.00	1.44 (0.99–2.09)	*−2*	1.83 (1.26–2.66)	*3*
Model 1 + hypertension	1.00	1.43 (0.99–2.08)	*−3*	1.81 (1.25–2.63)	*1*
Model 1 + high cholesterol^b^	1.00	1.45 (1.00–2.10)	*0*	1.79 (1.23–2.60)	*−1*
Model 1 + all risk factors	1.00	1.30 (0.89–1.89)	*−30*	1.57 (1.08–2.28)	*−24*
**CARDIOVASCULAR MORTALITY**					
Model 1^a^	1.00	1.66 (0.88–3.11)		1.95 (1.04–3.65)	
Model 1 + smoking	1.00	1.62 (0.86–3.04)	*−5*	1.87 (1.00–3.51)	*−6*
Model 1 + heavy drinking^b^	1.00	1.65 (0.88–3.09)	*−2*	1.91 (1.02–3.58)	*−3*
Model 1 + obesity^b^	1.00	1.58 (0.84–3.08)	*−10*	1.95 (1.04–3.65)	*0*
Model 1 + diabetes^b^	1.00	1.64 (0.84–2.97)	*−2*	1.99 (1.07–3.73)	*3*
Model 1 + hypertension^b^	1.00	1.62 (0.87–3.12)	*−4*	1.98 (1.05–3.69)	*2*
Model 1 + high cholesterol^b^	1.00	1.66 (1.00–2.10)	*1*	1.97 (1.03–3.60)	*2*
Model 1 + all risk factors	1.00	1.47 (0.78–2.77)	*−23*	1.82 (0.97–3.42)	*−11*

CI: Confidence Interval; HR: Hazard ratio; SES: Socioeconomic status; Δ: Difference.

a Sex- and year of birth-adjusted.

b Heavy drinking is defined as consuming ≥75 g of ethanol per week; obesity as body mass index ≥30 kg/m^2^; diabetes as fasting blood glucose ≥7.0 mmol/l (126 mg/dl) (1989, 2004) or positive glucosuria or history of diabetes (1994); hypertension as blood pressure≥140/90 mm Hg; high cholesterol as total cholesterol ≥6.2 mmol/l (240 mg/dl).

Results for CVD mortality and non-cancer/non-CVD mortality are presented in **Table 4**. The association of SES with cancer mortality did not reach statistical significance at conventional levels (HR = 1.44, 95%CI:0.76–2.75) and the contribution of risk factors to this association was thus not evaluated (results available upon request). Participants in the low vs. high SES groups had a greater risk of dying of cardiovascular mortality (HR = 1.95, 95% CI:1.04–3.65) and of non-cancer/non-CVD mortality (HR = 2.14, 95%CI: 1.10–4.16). Common lifestyle-related risk factors (smoking, heavy drinking, obesity, diabetes, hypertension, hypercholesterolemia) explained about 10% of the association between low SES and CVD mortality and about 20% of the association between low SES and non-cancer/non-CVD mortality.

**Table 4:** Socioeconomic differences in cardiovascular mortality (N = 3246, deaths = 219) and in non cancer non cardiovascular mortality (N = 3246, deaths = 171), and contribution of modifiable risk factors.

	Socioeconomic status				
	High	Middle		Low	
	HR (95% CI)	HR (95% CI)	% Δ	HR (95% CI)	% Δ
Model 1^a^	1.00	1.66 (0.88–3.11)		1.95 (1.04–3.65)	
Model 1 + smoking	1.00	1.62 (0.86–3.04)	*−5*	1.87 (1.00–3.51)	*−6*
Model 1 + heavy drinking^b^	1.00	1.65 (0.88–3.09)	*−2*	1.91 (1.02–3.58)	*−3*
Model 1 + obesity^b^	1.00	1.58 (0.84–3.08)	*−10*	1.95 (1.04–3.65)	*0*
Model 1 + diabetes^b^	1.00	1.64 (0.84–2.97)	*−2*	1.99 (1.07–3.73)	*3*
Model 1 + hypertension^b^	1.00	1.62 (0.87–3.12)	*−4*	1.98 (1.05–3.69)	*2*
Model 1 + high cholesterol^b^	1.00	1.66 (1.00–2.10)	*1*	1.97 (1.03–3.60)	*2*
Model 1 + all risk factors	1.00	1.47 (0.78–2.77)	*−23*	1.82 (0.97–3.42)	*−11*

CI: Confidence Interval; HR: Hazard ratio; SES: Socioeconomic status; Δ: Difference.

a Sex- and year of birth-adjusted.

b Heavy drinking is defined as consuming ≥75 g of ethanol per week; obesity as body mass index ≥30 kg/m^2^; diabetes as fasting blood glucose ≥7.0 mmol/l (126 mg/dl) (1989, 2004) or positive glucosuria or history of diabetes (1994); hypertension as blood pressure≥140/90 mm Hg; high cholesterol as total cholesterol ≥6.2 mmol/l (240 mg/dl).

### Sensitivity analysis

To make sure that cause-specific results were not affected by competing risks, we re-run our analysis for the SES-mortality association using a Fine-Gray model, allowing to study the relationship between covariates and cumulative incidences (via “subdistribution hazards”, i.e. treating the participants who died from another cause than the cause under study as if they were still alive). Results were similar to those reported in main analysis.

## Discussion

We found that SES (as measured by occupational position) was strongly associated with overall, CVD, and non-cancer/non-CVD mortality in the population of the Seychelles. A non significant trend was also found for cancer mortality. Common lifestyle-related risk factors explained a small proportion of social differences in mortality. This is one the first studies to examine social inequalities in cause-specific mortality in the African region.

This study shows disparities in mortality for chronic diseases such as CVD and cancer. There is currently a debate on whether actions to prevent the spread of NCDs in LMICs will benefit the “rich” more than the “poor” [46]. Our study suggests that NCDs disproportionally affect people with low SES, at least in a middle income country. This finding implies that prevention and control of NCDs should be tailored accordingly. Notably, the large social differences in mortality observed in this study arose despite the favorable social situation in Seychelles (free education and health care, social housing policy, price control of several essential foods, and high employment rates) comparing to most countries in the African region.

This is also one of the few studies to explore the role of the social distribution of lifestyle-related risk factors in shaping social differences in mortality in a middle income country. We found that a few major risk factors for chronic diseases (smoking, heavy drinking, and being obese or diabetic) explained a fairly small proportion of social differences in all-cause and CVD mortality, the main factor being smoking. Surprisingly, the contribution of lifestyle-related risk factors to social differences in mortality was smaller for CVD than for all-cause or non-cancer/non-CVD mortality. This may be related to the fact that heavy drinking, quite prevalent in the Seychelles[40], explained a substantial part of the SES gradient in all-cause and non-cancer/non-CVD mortality, while its contribution for CVD mortality was almost zero, as expected.

Several factors might explain the relatively small contribution of lifestyle-related risk factors to social differences in mortality in this study (10–20% compared to proportions of 20–75% often found in high income countries) [1]. First, risk factors were assessed only at a single point in time, while follow-up period could extend to more than 20 years for some participants; their contribution to the SES gradient in mortality might have been underestimated because of measurement error and changes of behaviors and biological risk markers over time[29]. Second, two main cardiovascular risk factors (diabetes and hypertension) were not patterned by SES in our study and hypercholesterolemia was more prevalent in high SES groups. Third, the contribution of two important risk factors for chronic diseases, physical activity and dietary patterns, were not evaluated because of lack of this information in the three surveys. Finally, the social patterning of lifestyle-related risk factors was relatively weak in this study, suggesting that other factors (for example living/working conditions, cultural or psycho-social factors) might be driving social inequalities in mortality in this population. Further studies should examine other potential mechanisms explaining social differences in mortality in Seychelles.

### Strengths and limitations

This is one of the first studies in the African region, and one of the few in LMICs, to use population-based data for examining socioeconomic differences in all-cause and cause-specific mortality. This study benefited from the availability of data on several exposures arising from population-based surveys (e.g. lifestyles, blood tests), reliance on a same methodology over time, and reliable causes of death derived from medically certified death certificates.

This study also has some limitations. First, because of the fairly small number of deaths, we could not examine mortality according to age or other categories. For the same reason, we could not examine cancer mortality separately by cancer site. The fact that the direction of the association between SES and cancer mortality is generally found to differ by cancer site might explain the lack of association between SES and cancer mortality in this study. Second, it can sometimes be difficult to ascertain the main cause of death among the elderly, because of multimorbidity and for deaths occurring outside of a hospital. These problems were minimized in this study because main paraclinical investigations were available throughout the study period; a substantial proportion of deaths occurring outside of a hospital underwent autopsy; and free health care in Seychelles reduces access barriers and improves conditions for adequate diagnosis. Third, a potential limitation is related to the fact that certain deaths could have been missed (and thus considered as alive on December 31^st^, 2012) when linking survey data with vital statistics, e.g. if participants left the country or changed their names. This would have lead to an overestimation of the probability of survival, but not necessarily to a bias in the estimated hazard ratios. Also, survival analysis for specific causes of death is complicated by the issue of competing risks. In Table 4, we have assessed the relationship between various covariates and cause-specific hazards. In a context of competing risks, however, a cause-specific hazard is not one-to-one related to the probability (or cumulative incidence) of dying from this cause, and the way covariates are associated with the former may differ from the way they are associated with the latter (see e.g.[47]). Fourth, although we controlled our analysis for estimating the contribution of common lifestyle-related risk factors to the SES-mortality association for known confounders (ie: age and gender), we cannot exclude the presence of unmeasured mediator-outcome or exposure-outcome confounders[48]. Finally, although we recognize that SES is a multifaceted concept involving different dimensions (from resources to prestige), in this study we only used occupational position as the indicator of SES. This measure has been extensively used in social epidemiology[49] and provides a valid approximation of SES in settings with high employment rates such as the Seychelles. Fairly similar distributions of SES based on occupation were found across surveys, in contrast, for example, to changing distributions of education categories across successive surveys, consistent with a large secular increase in the mean number of school years.

More generally, the situation in Seychelles, which has now become an upper middle income country, does not reflect the predominant social and health conditions of several other countries in the region. Yet, because most countries in the African region lack vital statistics with coverage at the entire population level or otherwise reliable mortality data, our study provides an important account. It is important that future studies in the region, for example using existing cohorts [50], [51], examine social inequalities in cause-specific mortality in settings characterized by less favorable socioeconomic conditions and at an earlier stage of the health transition.

## Conclusions

In one of the first population-based studies to assess social inequalities in cause-specific mortality in the African region, low SES (as measured by occupational position) strongly predicted overall and cardiovascular mortality. Major risk factors for chronic diseases, particularly smoking, explained part of this association. Our findings support the view that the burden of NCDs may disproportionally affect people with a low SES in LMICs and suggest that interventions to prevent and control NCDs should be tailored accordingly.
